# Supplementary material for: Hypoxanthine phosphoribosyl transferase 1 metabolizes temozolomide to activate AMPK for driving chemoresistance of glioblastomas
Source: Nat Commun. 2023 Sep 22;14:5913. doi: 10.1038/s41467-023-41663-2 (PMC10516874; doi:10.1038/s41467-023-41663-2)
Supplement: Supplementary file 1 — Supplementary Information [file 41467_2023_41663_MOESM1_ESM.pdf]

SUPPLEMENTAL INFORMATION

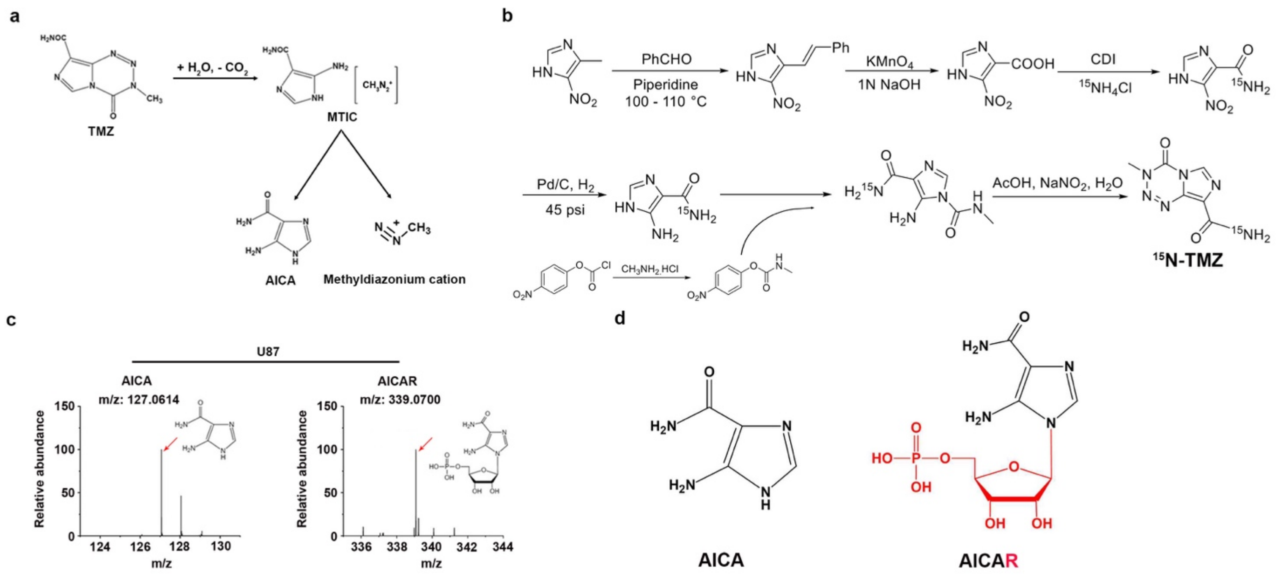

Supplementary Fig. 1 AICAR derived from AICA is a *bona fide* intracellular metabolite of TMZ

a Scheme of TMZ metabolism.

b Scheme of ^{15}N-TMZ synthesis.

c Representative tandem mass spectra of AICA and AICAR.

d The chemical structural formulas of AICA and AICAR. Red indicates the difference between AICA and AICAR.

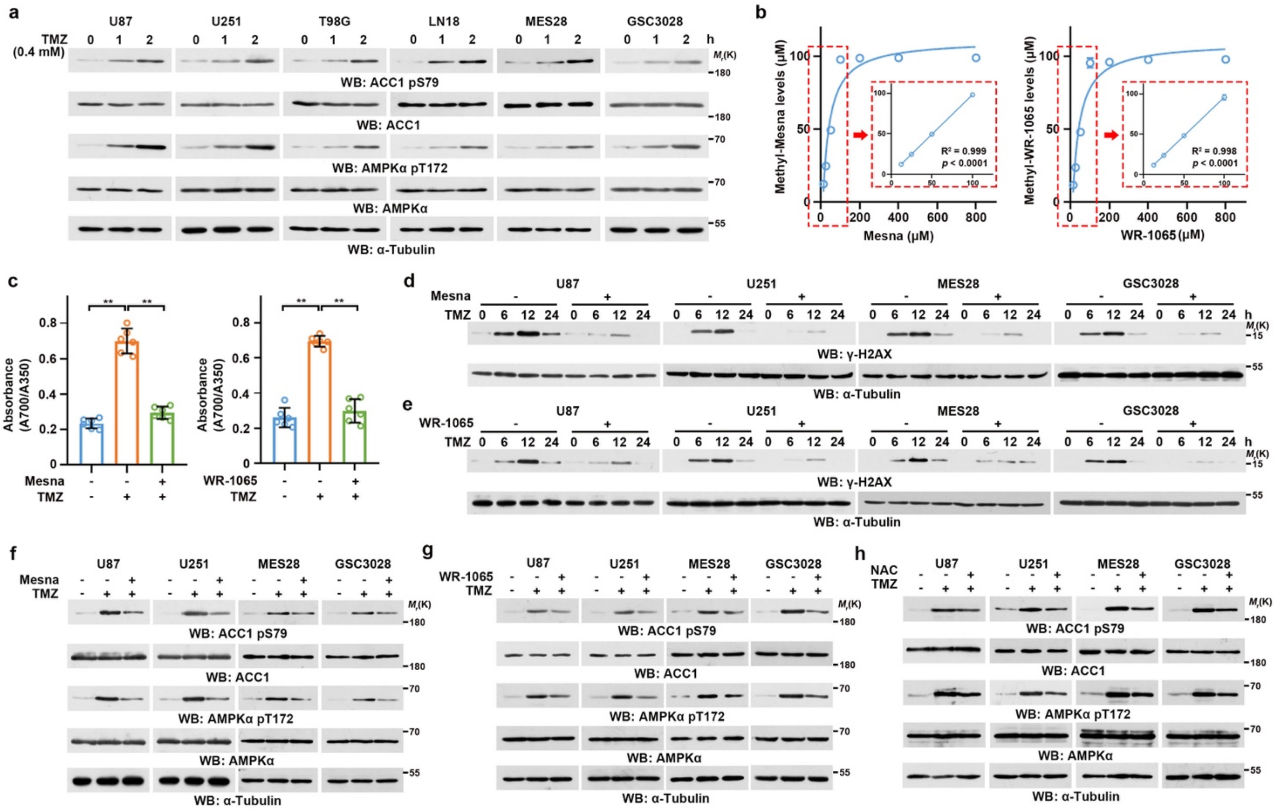

Supplementary Fig. 2 TMZ-derived AICA activates AMPK

a, d-h Immunoblotting analyses were performed with the indicated antibodies. Three biological repeats were repeated independently with similar results.

a The indicated cells were treated with 0.2 mM of TMZ for the indicated time points.

b Serial dosages of Mesna (12, 25, 50, 100, 200, 400, 800 μM) or WR-1065 (12, 25, 50, 100, 200, 400, 800 μM) were incubated with TMZ (100 μM) for 5 min. HPLC-MS analyses was used to measure methyl-Menas and methyl-WR-1065 production. Mesna vs methyl-Mesna, P = 1.62e-08; WR-1065 vs methyl-WR-1065, P = 2.37e-08.

c U87 cells pretreated with or without 10 μM Mesna or 40 μM WR-1065, were treated with or without 0.2 mM of TMZ for 30 min. Genomic DNA (100 ng) from U87 cells was extracted. Intracellular O6-mG levels were measured using ExBenzi nanoprobes and detection probes. Data represent the mean ± SD from sextuplicate experiments. **, P < 0.001. Left, Control vs TMZ, P = 3.36e-08; TMZ vs Mesna + TMZ, P = 1.78-07; Right, Control vs TMZ, P = 1.17e-08; TMZ vs WR-1065 + TMZ, P = 1.13-07.

d and **e**, The indicated cells pretreated with or without 10 μM Mesna (**d**) or 40 μM WR-1065 (**e**), were treated with or without 0.2 mM of TMZ for the indicated time points.

f-h The indicated cells pretreated with or without 10 μM Mesna (**f**), 40 μM WR-1065 (**g**), or 2 mM NAC (**h**) were treated with or without 0.2 mM of TMZ for 2 h.

Statistics: **b** Two-sided Student's t test for two-group comparison; **c** unpaired Student's t test for two-

group comparison. Source data are provided as a Source Data file.

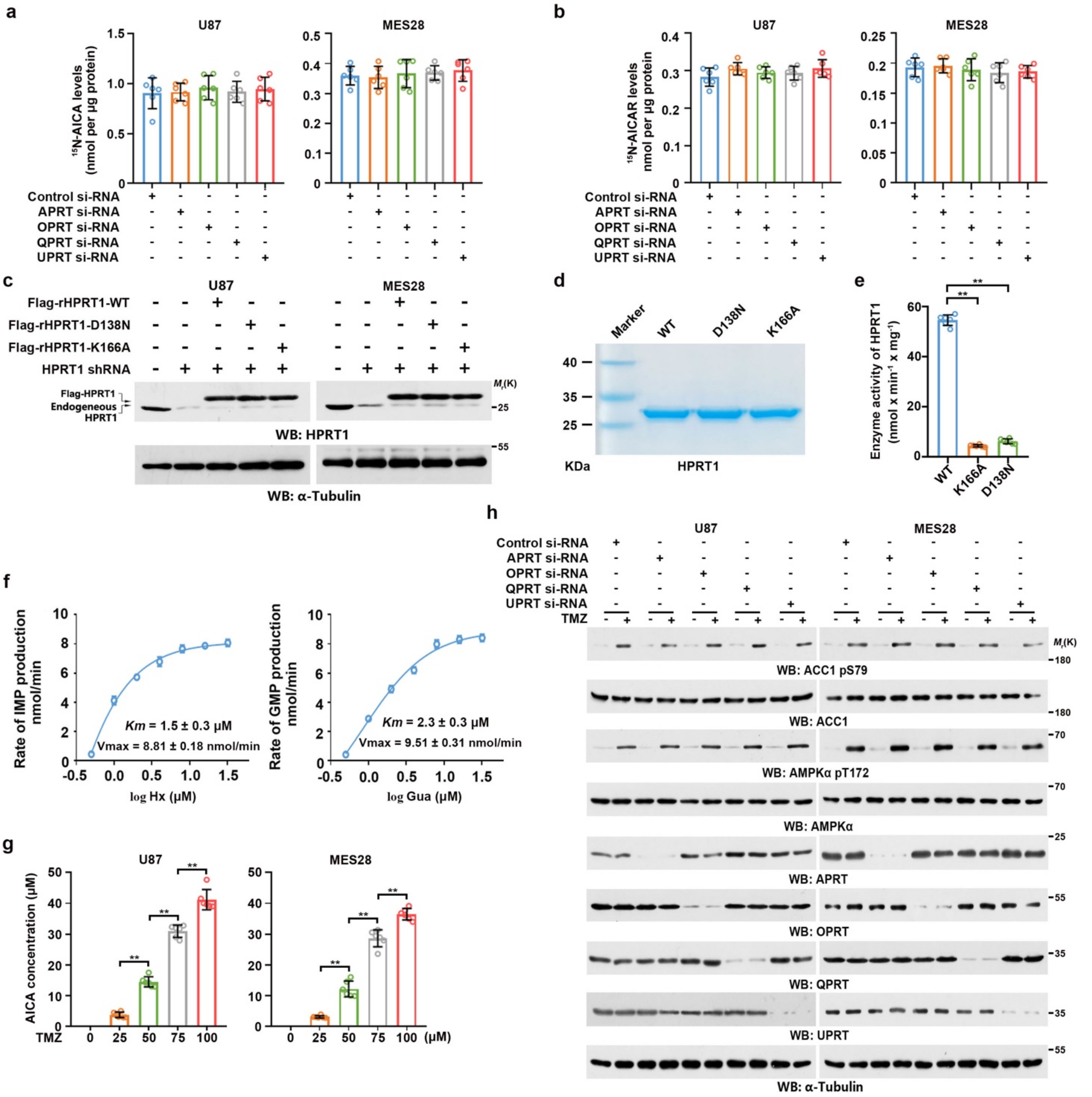

Supplementary Fig. 3 HPRT1 converts TMZ-derived AICA into AICAR

a and **b** Cells were transfected with APRT, OPRT, QPRT, and UPRT siRNA. Intracellular ^{15}N-AICA (**a**) and ^{15}N-AICAR (**b**) were measured by HPLC-MS. Data represent the mean ± SD from sextuplicate experiments.

c Cells with or without HPRT1 depletion were reconstituted with or without WT Flag-HPRT1, Flag-HPRT1 D138N or Flag-HPRT1 K166A mutant. Immunoblotting analyses were performed with the indicated antibodies. Three biological repeats were repeated independently with similar results.

d Coomassie brilliant blue image of purified WT His-HPRT1, His-HPRT1 D138N and K166A mutant proteins. Three biological repeats were repeated independently with similar results.

e WT His-HPRT1, His-HPRT1 D138N, and K166A mutant proteins were incubated with hypoxanthine for 2 h, followed by HPLC-MS analysis. HPRT1 activity was measured according to IMP production. Data represent the mean ± SD from sextuplicate experiments. **, P < 0.001. WT vs K166A, P = 6.64e-14; WT vs D138N, P = 2.03e-13.

f Michaelis-Menten curve of HPRT1 for hypoxanthine (left) and guanine (right). Reactions were performed by mixing purified active HPRT1 and the indicated substrates. *K*m, the Michaelis constant; Vmax, the maximum reaction rate. Data represent the mean ± SD from sextuplicate experiments. **, P < 0.001. Hx, hypoxanthine; Gua, guanine.

g Cells were treated with the indicated concentration of TMZ for 2 h. Intracellular AICA was measured by HPLC-MS. Data represent the mean ± SD from sextuplicate experiments. **, P < 0.001. U87, 25 vs 50 μM, P = 6.98e-08; 50 vs 75 μM, P = 2.43e-08; 75 vs 100 μM, P = 6.76e-05; MES28, 25 vs 50 μM, P = 6.6e-06; 50 vs 75 μM, P = 8.27e-07; 75 vs 100 μM, P = 0.00017.

h Cells were transfected with APRT, OPRT, QPRT, and UPRT siRNA, followed by immunoblotting analyses with the indicated antibodies. Three biological repeats were repeated independently with similar results.

Statistics: **e, g** unpaired Student's t test for two-group comparison. Source data are provided as a Source Data file.

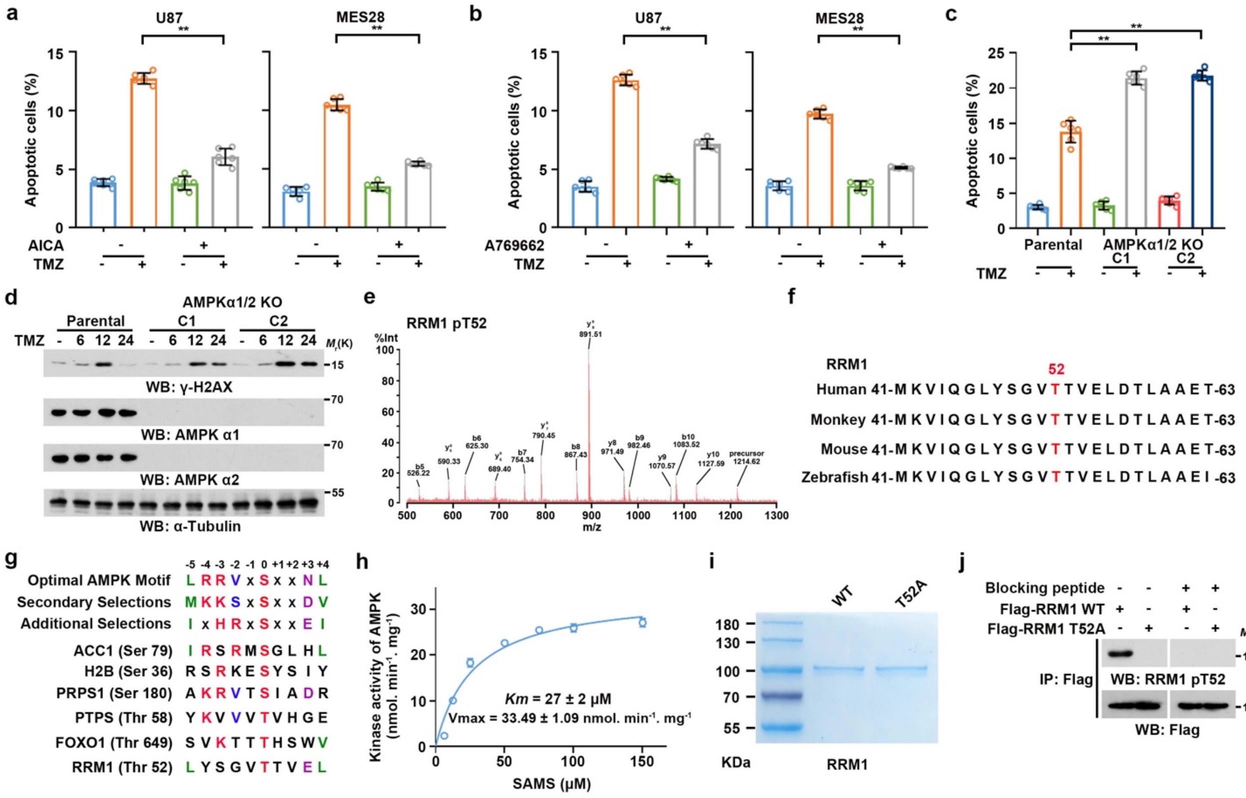

Supplementary Fig. 4 TMZ-activated AMPK phosphorylates RRM1 at T52 to promote DNA damage repair

a Cells pretreated with or without 0.25 mM of AICA were treated with 0.2 mM of TMZ for 24 h. The rate of apoptotic cells was examined by FACS. Data represent the mean ± SD from sextuplicate experiments. **, P < 0.001. U87, TMZ vs AICA + TMZ, P = 2.89e-09; MES28, TMZ vs AICA + TMZ, P = 4.85e-10.

b Cells pretreated with or without 0.25 mM of A769662 were treated with 0.2 mM of TMZ for 24 h. The rate of apoptotic cells was examined by FACS. Data represent the mean ± SD from sextuplicate experiments. **, P < 0.001. U87, TMZ vs A769662 + TMZ, P = 1e-09; MES28, TMZ vs A769662 + TMZ, P = 8.31e-11.

c AMPKα1/2 DKO U87 cells were treated with 0.2 mM of TMZ for 24 h. The rate of apoptotic cells was examined by FACS. Data represent the mean ± SD from sextuplicate experiments. **, P < 0.001. Parental + TMZ vs C1 + TMZ, P = 1.2e-06; Parental + TMZ vs C2 + TMZ, P = 4.82e-07.

d AMPKα1/2 DKO U87 cells were treated with or without 0.2 mM of TMZ for the indicated time points. Immunoblot analyses were performed with the indicated antibodies. C1, C2, two clones of AMPKα1/2 DKO U87 cells. Three biological repeats were repeated independently with similar results.

e Flag-RRM1 was enriched in TMZ-treated U251 cells and analyzed by mass spectrometry. Mass spectrometric analysis was performed on a tryptic fragment at m/z 891.51 Da (mass error was +5.55

ppm) matched to the charged peptide 49-SGVTTVELDTL-59; the results suggested that T52 was phosphorylated.

f Alignment of the RRM1 protein sequence spanning T52 in different species.

g Alignment of RRM1 T52 to the optimal AMPK motif. The AMPK recognition motif prefers hydrophobic residues at the -5 position and +4 position, basic residues (R or K) at the -3 and -4 positions, and polar residues (D or N) at the +3 position relative to the phosphoacceptor site (S or T). In addition, a strong bias is exhibited toward serine or valine at the -2 position. The reported AMPK substrates (ACC1, H2B, PRPS1, PTPS, and FOXO1) are shown.

h Validation of AMPK activity using SAMS peptide as substrates. Data represent the mean ± SD from triplicate experiments. **, $P < 0.001$.

i Coomassie brilliant blue image of purified His-RRM1 WT and His-RRM1 T52A mutant proteins. Three biological repeats were repeated independently with similar results.

j RRM1 T52 phosphorylation-specific antibody was validated. Purified Flag-PTEN WT or Flag-RRM1 T52A was incubated with active AMPK and subjected for immunoblotting analyses with anti-RRM1 T52 phosphorylation antibody in the presence or absence of phosphorylated RRM1 peptides (aa 45-57). Three biological repeats were repeated independently with similar results.

Statistics: **a-c** unpaired Student's t test for two-group comparison. Source data are provided as a Source Data file.

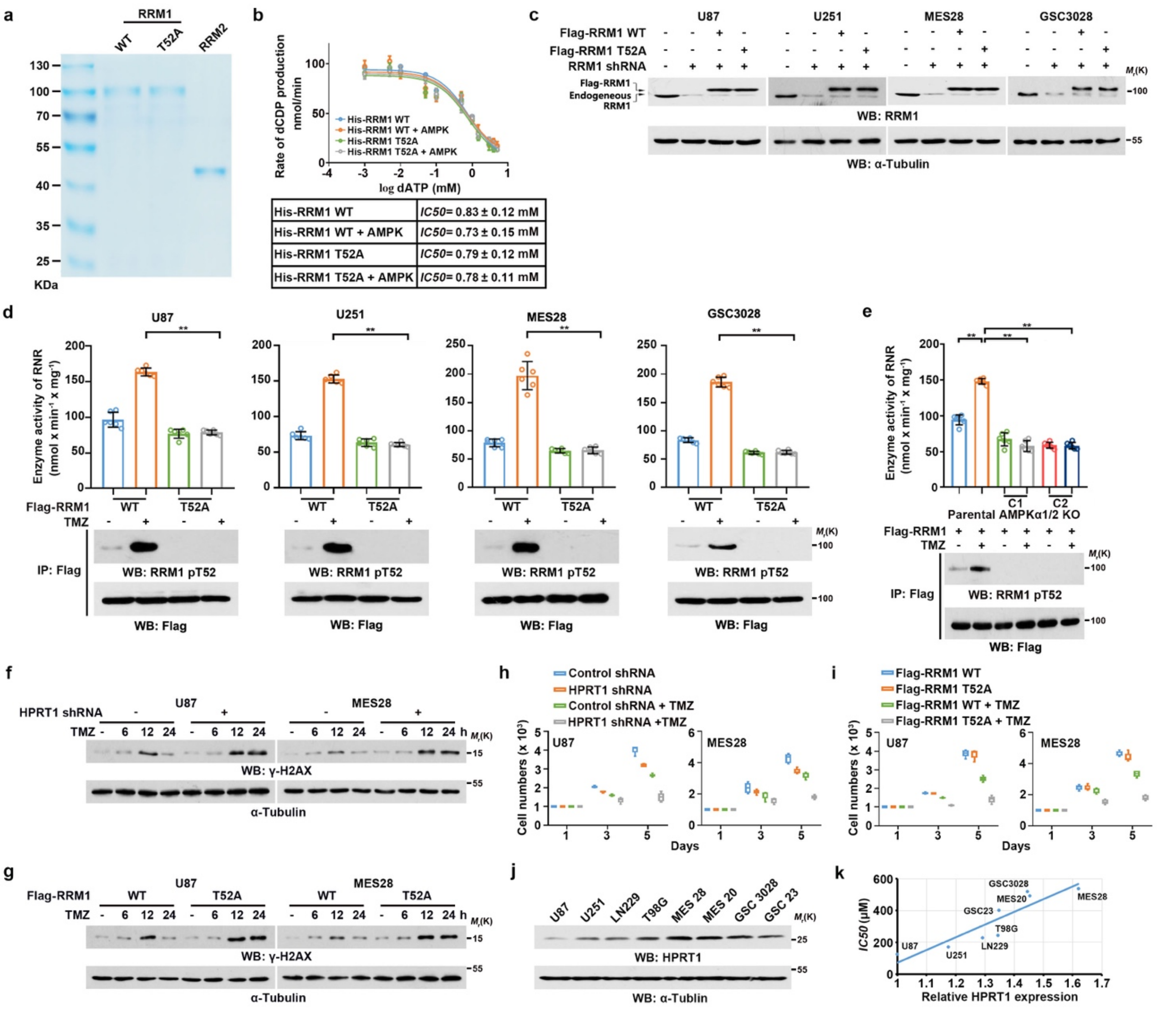

Supplementary Fig. 5 TMZ-activated AMPK phosphorylates RRM1 at T52 to promote DNA damage repair

a Coomassie brilliant blue image of purified WT His-RRM1, His-RRM1 T52A and His-RRM2 proteins. Three biological repeats were repeated independently with similar results.

b His-RRM1 WT or His-RRM1 T52A mutant proteins were incubated with active AMPK and His-RRM2 in the presence of dATP for 20 min, followed by HPLC-MS analysis. The RNR activity was measured according to dCDP production. Data represent the mean ± SD from sextuplicate experiments.

c The indicated cells with or without RRM1 shRNA expression were reconstituted with or without WT Flag-RRM1 or Flag-RRM1 T52A expression. Immunoblotting analyses were performed with the indicated antibodies. Three biological repeats were repeated independently with similar results.

d The indicated cells with reconstituted expression of WT Flag-RRM1 or the Flag-RRM1 T52A mutant were treated with or without 0.2 mM of TMZ for 2 h. The RNR activity was measured according to dCDP production. Data represent the mean ± SD from sextuplicate experiments. **, $P < 0.001$.

e Parental or AMPKα1/2 DKO U87 cells expressing Flag-RRM1 were treated with or without 0.2 mM of TMZ for 2 h. RNR activity was measured according to dCDP production. Immunoblotting analysis was performed to confirm the AMPK-mediated phosphorylation status of RRM1. Data represent the mean ± SD from sextuplicate experiments. **, $P < 0.001$.

f Cells with or without HPRT1 depletion were treated with or without 0.2 mM of TMZ for the indicated time courses. Immunoblotting analyses were performed with the indicated antibodies. Three biological repeats were repeated independently with similar results.

g RRM1-depleted cells with reconstituted expression of WT Flag-RRM1 or Flag-RRM1 T52A mutant were treated with or without 0.2 mM of TMZ for the indicated time courses. Immunoblotting analyses were performed with the indicated antibodies. Three biological repeats were repeated independently with similar results.

h Cells with or without HPRT1 depletion were treated with or without 0.2 mM of TMZ for the indicated time points. The growth of cells was examined. Data represent the mean ± SD from sextuplicate experiments. Box plots indicate the median (centre line), the first and third quartiles (box limits). The whiskers indicate the maxima and minima.

i RRM1-depleted cells with reconstituted expression of WT Flag-RRM1 or Flag-RRM1 T52A mutant were treated with or without 0.2 mM of TMZ for the indicated time points. The growth of cells was examined. Data represent the mean ± SD from sextuplicate experiments. Box plots indicate the median (centre line), the first and third quartiles (box limits). The whiskers indicate the maxima and minima.

j The expression of HPRT1 in the indicated GBM cells was measured by immunoblotting analysis with the indicated antibodies. Three biological repeats were repeated independently with similar results.

k The correlation between HPRT1 expression and the IC50 of the indicated cells toward TMZ was analyzed using Spearman rank correlation analysis.

Source data are provided as a Source Data file.

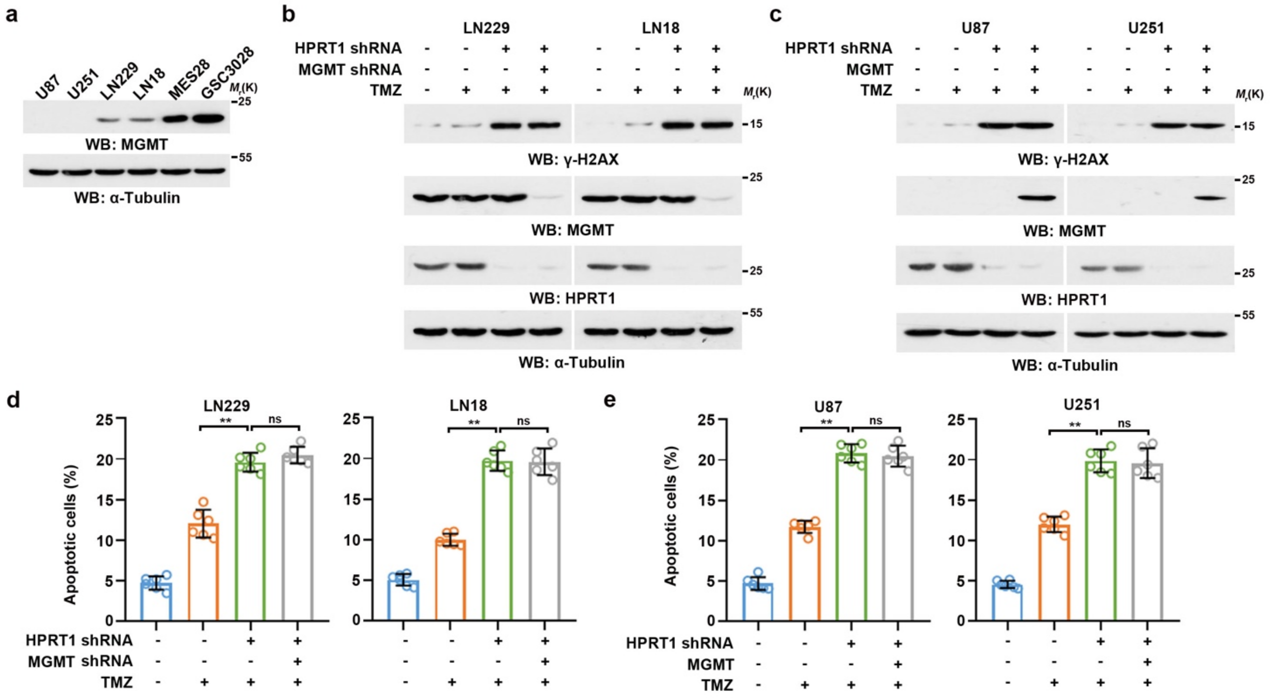

Supplementary Fig. 6 MGMT is not relevant to TMZ/HPRT1-mediated DNA damage response

a The expression of MGMT in the indicated cells was measured by immunoblotting analysis with the indicated antibodies. Three biological repeats were repeated independently with similar results.

b and **d** MGMT-intact LN229 and LN18 cells with or without depletion of HPRT1 or MGMT were treated with or without TMZ (0.2 mM) for 48 h. (**b**) Immunoblotting analyses were performed with the indicated antibodies. Three biological repeats were repeated independently with similar results. (**d**) The rate of apoptotic cells was examined by FACS. Data represent the mean ± SD from sextuplicate experiments. **, P < 0.001. LN229, TMZ vs HPRT1 shRNA + TMZ, P = 4.37e-06; HPRT1 shRNA + TMZ vs HPRT1 shRNA + MGMT shRNA + TMZ, P = 0.2. LN18, TMZ vs HPRT1 shRNA + TMZ, P = 1.48e-08; HPRT1 shRNA + TMZ vs HPRT1 shRNA + MGMT shRNA + TMZ, P = 0.87.

c and **e** MGMT-null U87 and U251 cells with or without HPRT1 depletion were co-transfected with or without MGMT, followed by TMZ (0.2 mM) treatment for 48 h. (**c**) Immunoblotting analyses were performed with the indicated antibodies. Three biological repeats were repeated independently with similar results. (**e**) The rate of apoptotic cells was examined by FACS. Data represent the mean ± SD from sextuplicate experiments. **, P < 0.001. U87, TMZ vs HPRT1 shRNA + TMZ, P = 1.53e-08; HPRT1 shRNA + TMZ vs HPRT1 shRNA + MGMT + TMZ, P = 0.63. U251, TMZ vs HPRT1 shRNA + TMZ, P = 5.11e-07; HPRT1 shRNA + TMZ vs HPRT1 shRNA + MGMT + TMZ, P = 0.77.

Statistics: **d, e** unpaired Student's t test for two-group comparison. Source data are provided as a Source Data file.

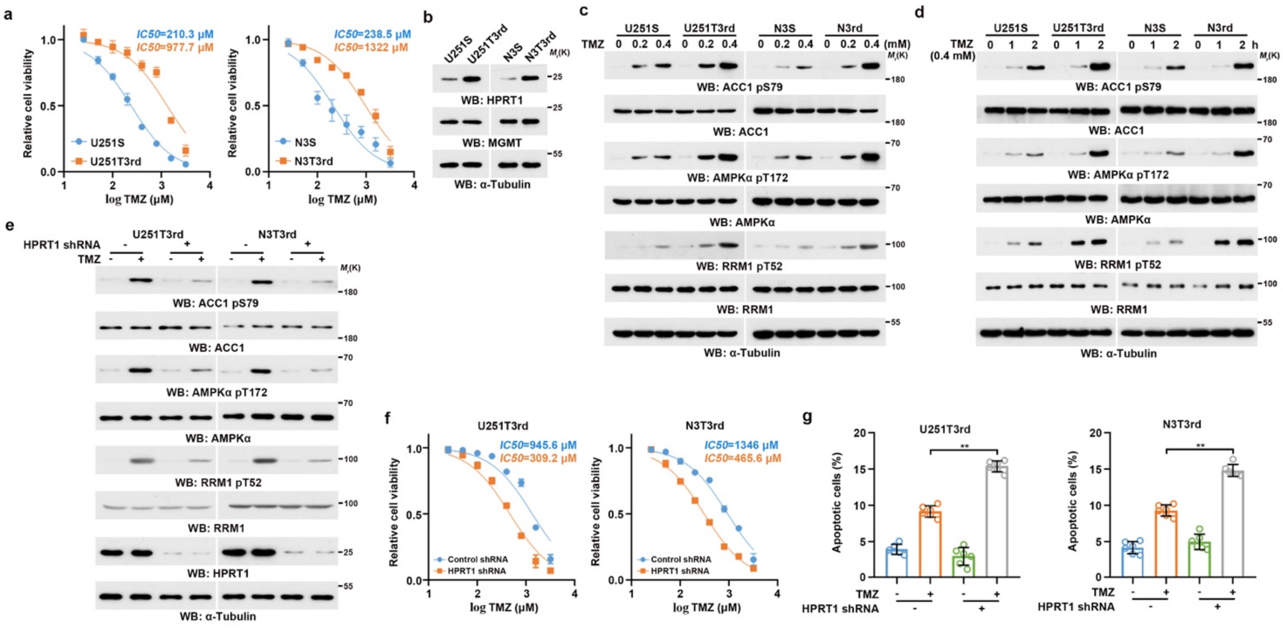

Supplementary Fig. 7 HPRT1 mediates the sensitivity of TMZ in TMZ-resistant GBM cells

b-e Immunoprecipitation and immunoblot analyses were performed with the indicated antibodies. Three biological repeats were repeated independently with similar results.

a Dose-response curves of TMZ treatment in TMZ-resistant cell lines and their paired sensitive counterparts (U251T3rd vs. U251S, and N3T3rd vs. N3S). IC50 of TMZ for the indicated cells was measured using nonlinear regression analysis of the dose-response curves. Data represent the mean ± SD from triplicate experiments. **, P < 0.001.

b TMZ-resistant cell lines and their paired sensitive counterparts were collected.

c Cells were treated with the indicated dose of TMZ for 2 h.

d Cells were treated with 0.4 mM of TMZ for the indicated time course.

e Cells with or without HPRT1 depletion were treated with or without 0.2 mM of TMZ for 2 h.

f Dose-response curves of TMZ treatment in cells with or without HPRT1 depletion. IC50 of TMZ for the indicated cells was measured using nonlinear regression analysis of the dose-response curves. Data represent the mean ± SD from triplicate experiments. **, P < 0.001.

g Cells with or without HPRT1 depletion were treated with or without 0.2 mM of TMZ for 24 h, The rate of apoptotic cells was examined by FACS. Data represent the mean ± SD from sextuplicate experiments. **, P < 0.001. U251T3rd, TMZ vs HPRT1 shRNA + TMZ, P = 6.58e-08; N3T3rd, TMZ vs HPRT1 shRNA + TMZ, P = 3.2e-07.

Source data are provided as a Source Data file.

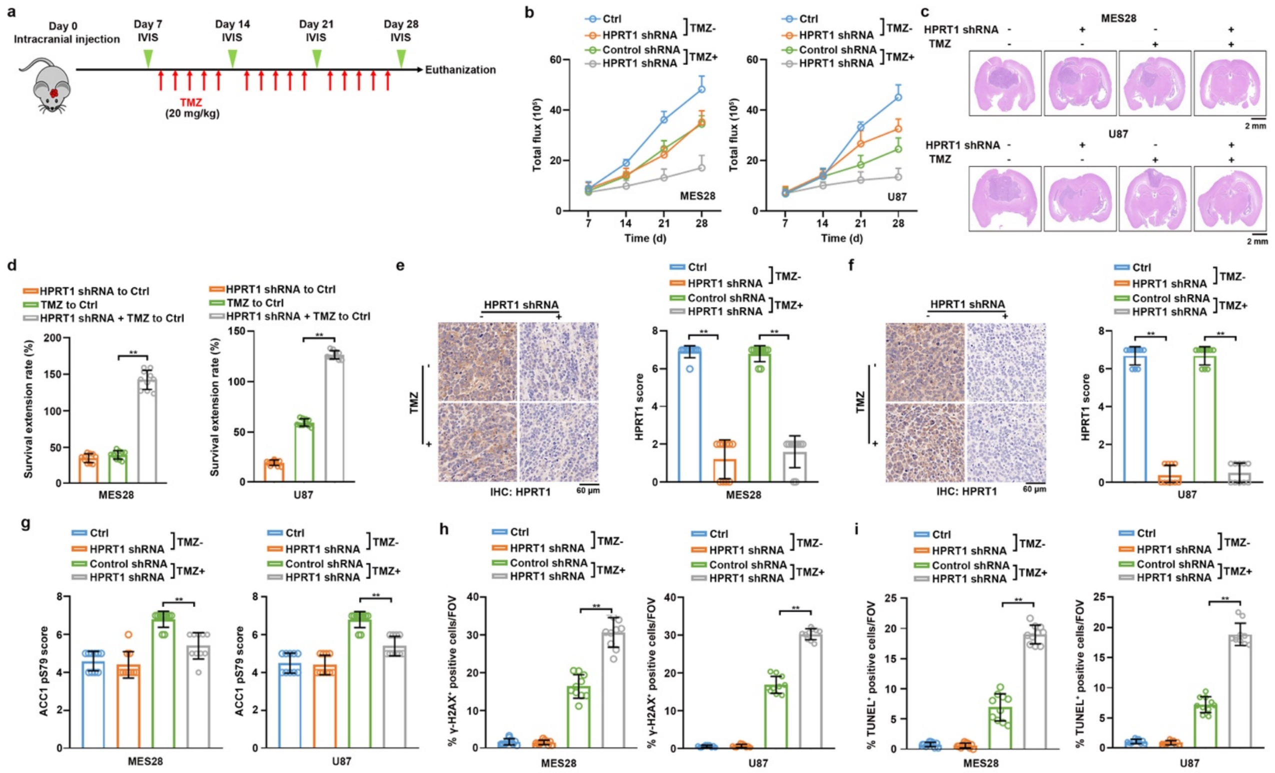

Supplementary Fig. 8 Inhibition of HPRT1-mediated RRM1 T52 phosphorylation sensitizes brain tumors to TMZ treatment

a Schematic diagram of the treatment of TMZ in tumor-bearing mice. TMZ (20 mg/kg) was given for 5 consecutive days since Day 7 after tumor implantation.

b Luciferase-expressing MES28 and U87 cells with or without HPRT1 depletion were intracranially injected into nude mice (n = 10 for each group). Shown were the curve of luminescence intensity at the indicated time points.

c Representative H&E-stained brain sections of the indicated groups of mice were shown. Scale bars, 2 mm. (n = 10 for each group).

d Survival extension of mice with the indicated treatments were calculated. (n = 10 for each group). **, P < 0.001. MES28, TMZ to Ctrl vs HPRT1 shRNA + TMZ to Ctrl, P = 1.13e-14; U87, TMZ to Ctrl vs HPRT1 shRNA + TMZ to Ctrl, P = 9.38e-19.

e and **f** Representative IHC images and the quantification of HPRT1 were shown. (n = 10 for each group). Scale bars, 60 μm. **, P < 0.001. MES28, Ctrl vs HPRT1 shRNA, P = 2.13e-12; Control shRNA + TMZ vs HPRT1 shRNA + TMZ, P = 1e-12; U87, Ctrl vs HPRT1 shRNA, P = 2.42e-16; Control shRNA + TMZ vs HPRT1 shRNA + TMZ, P = 3.9e-16.

g Quantification of ACC1 pS79 levels were shown. (n = 10 for each group). **, P < 0.001. MES28, Control shRNA + TMZ vs HPRT1 shRNA + TMZ, P = 3.75e-05; U87, Control shRNA + TMZ vs HPRT1 shRNA + TMZ, P = 3.11e-06.

h Quantification of γ-H2AX levels were shown. (n = 10 for each group). **, $P < 0.001$. MES28, Control shRNA + TMZ vs HPRT1 shRNA + TMZ, P = 4.16e-08; U87, Control shRNA + TMZ vs HPRT1 shRNA + TMZ, P = 5.61e-12.

i Quantification of TUNEL were shown. (n = 10 for each group). **, $P < 0.001$. MES28, Control shRNA + TMZ vs HPRT1 shRNA + TMZ, P = 3.93e-11; U87, Control shRNA + TMZ vs HPRT1 shRNA + TMZ, P = 3.5e-12.

Statistics: **d-i** unpaired Student's t test for two-group comparison. Source data are provided as a Source Data file.

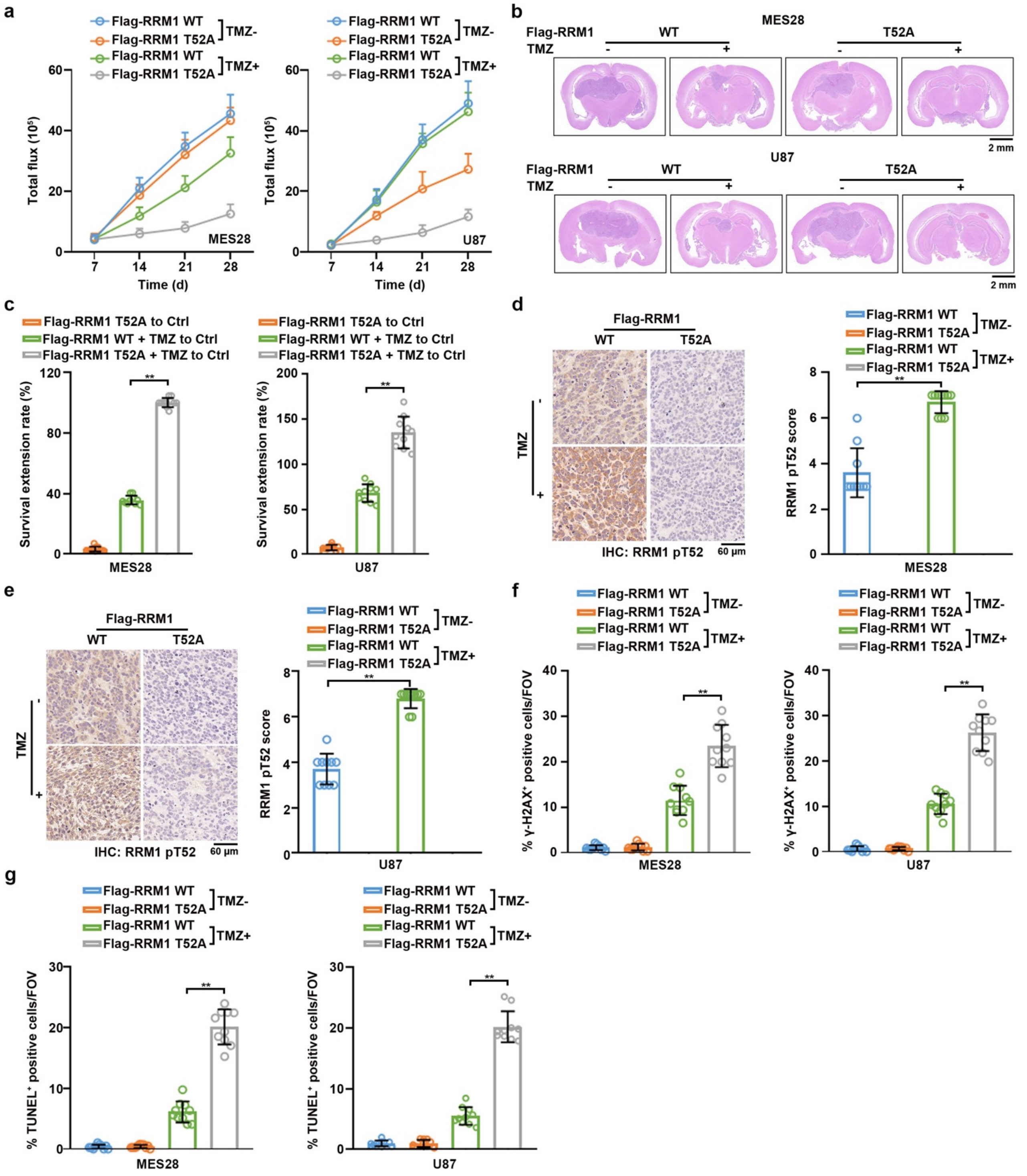

Supplementary Fig. 9 Expression of RRM1 T52A sensitizes brain tumors to TMZ treatment

a Luciferase-expressing MES28 and U87 cells with reconstituted expression of WT Flag-RRM1 or the Flag-RRM1 T52A mutant were intracranially injected into nude mice (n = 10 for each group). Shown were the curve of luminescence intensity at the indicated time points.

b Representative H&E-stained brain sections of the indicated groups of mice were shown. Scale bars, 2 mm.

c Survival extension of mice with the indicated treatments were calculated. (n = 10 for each group). **, P < 0.001. MES28, Flag-RRM1 WT + TMZ to Ctrl vs Flag-RRM1 T52A + TMZ to Ctrl, P = 1.19e-20; U87, Flag-RRM1 WT + TMZ to Ctrl vs Flag-RRM1 T52A + TMZ to Ctrl, P = 3.93e-09.

d and **e** Representative IHC images and the quantification of RRM1 pT52 were shown. (n = 10 for each group). Scale bars, 60 μm. **, P < 0.001. MES28, Flag-RRM1 WT vs Flag-RRM1 WT + TMZ, P = 1.4e-07; U87, Flag-RRM1 WT vs Flag-RRM1 WT + TMZ, P = 3.3e-10.

f Quantification of γ-H2AX were shown. (n = 10 for each group). **, P < 0.001. MES28, Flag-RRM1 WT + TMZ vs Flag-RRM1 T52A + TMZ, P = 2.85e-06; U87, Flag-RRM1 WT + TMZ vs Flag-RRM1 T52A + TMZ, P = 2.92e-09.

g Quantification of TUNEL were shown. (n = 10 for each group). **, P < 0.001. MES28, Flag-RRM1 WT + TMZ vs Flag-RRM1 T52A + TMZ, P = 1.1e-10; U87, Flag-RRM1 WT + TMZ vs Flag-RRM1 T52A + TMZ, P = 5.55e-12.

Statistics: **c-g** unpaired Student's t test for two-group comparison. Source data are provided as a Source Data file.

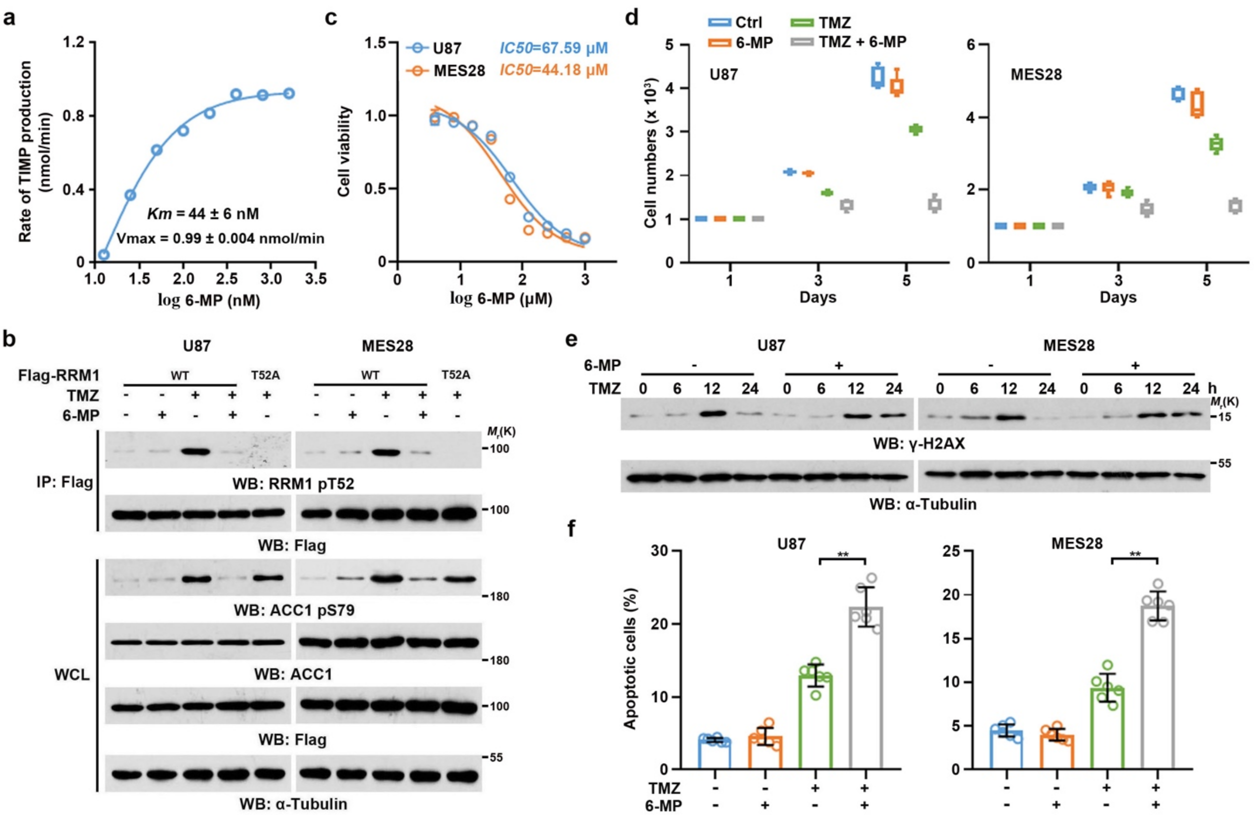

Supplementary Fig. 10 6-MP sensitizes GBM cells to TMZ treatment

a Michaelis-Menten curve of HPRT1 for 6-MP. Reactions were performed by mixing purified active HPRT1 and 6-MP. Data represent the mean ± SD from sextuplicate experiments.

b Cells were treated with TMZ (0.2 mM) together with or without 6-MP (10 μM) for the indicated time points. Immunoblot analyses were performed with the indicated antibodies. Three biological repeats were repeated independently with similar results.

c Dose-response curves of 6-MP treatment in U87 and MES28. IC50 of 6-MP for U87 and MES28 was measured using nonlinear regression analysis of the dose-response curves, respectively. Data represent the mean ± SD from triplicate experiments.

d Cells were treated with TMZ (0.2 mM), 6-MP (10 μM), or a combination of TMZ and 6-MP for the indicated time points. Cell numbers were counted at the indicated time points. Data represent the mean ± SD from quintuplicate experiments. Box plots indicate the median (centre line), the first and third quartiles (box limits). The whiskers indicate the maxima and minima.

e Cells were treated with TMZ (0.2 mM) together with or without 6-MP (10 μM) for the indicated time points. Immunoblot analyses were performed with the indicated antibodies. Three biological repeats were repeated independently with similar results.

f Cells were treated with TMZ (0.2 mM), 6-MP (10 μM) or a combination of TMZ and 6-MP for 24 h. The rate of apoptotic cells was examined by FACS. Data represent the mean ± SD from sextuplicate

experiments. **, $P < 0.001$. U87, TMZ vs TMZ + 6-MP, $P = 2.26e-05$; MES28, TMZ vs TMZ + 6-MP, $P = 1.61e-06$.

Statistics: **f** unpaired Student's t test for two-group comparison. Source data are provided as a Source Data file.

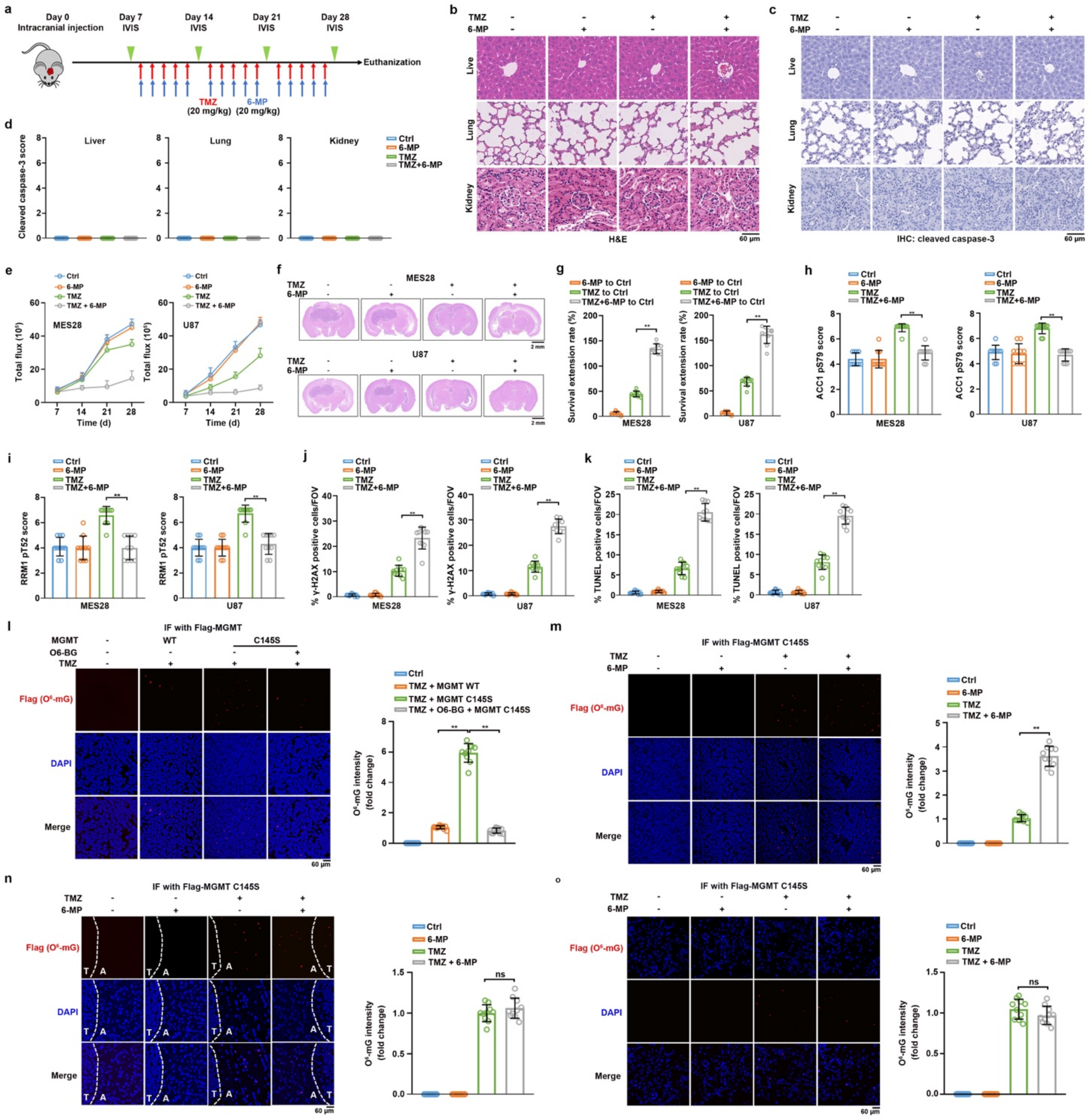

Supplementary Fig. 11 6-MP sensitizes brain tumors to TMZ treatment

a Schematic diagram of the treatment of TMZ with or without 6-MP in tumor-bearing mice. TMZ (20 mg/kg) and 6-MP (20 mg/kg) were given for 5 consecutive days since Day 7 after tumor implantation.

b Representative H&E images of liver, lung, and kidney tissues of mice treated with or without TMZ or 6-MP. Scale bars, 60 μm. (n = 10 for each group).

c and **d** Representative IHC images (**c**) and the quantification (**d**) of Cleaved caspase-3 in liver, lung, and kidney tissues were shown. Scale bars, 60 μm. (n = 10 for each group).

e Luciferase-expressing MES28 and U87 cells were intracranially injected into nude mice (n = 10 for each group). Shown were the curve of luminescence intensity at the indicated time points.

f Representative H&E-stained brain sections of the indicated groups of mice were shown. Scale bars, 2 mm. (n = 10 for each group).

g Survival extension of mice with the indicated treatments were calculated. (n = 10 for each group). **, $P < 0.001$. MES28, TMZ to Ctrl vs TMZ + 6-MP to Ctrl, P = 1.78e-15; U87, TMZ to Ctrl vs TMZ + 6-MP to Ctrl, P = 7.83e-12.

h Quantification of ACC1 pS79 were shown. (n = 10 for each group). **, $P < 0.001$. MES28, TMZ vs TMZ + 6-MP, P = 1.35e-08; U87, TMZ vs TMZ + 6-MP, P = 5.19e-09.

i Quantification of RRM1 pT52 were shown. (n = 10 for each group). **, $P < 0.001$. MES28, TMZ vs TMZ + 6-MP, P = 1.53e-06; U87, TMZ vs TMZ + 6-MP, P = 1.21e-06.

j Quantification of γ-H2AX were shown. (n = 10 for each group). **, $P < 0.001$. MES28, TMZ vs TMZ + 6-MP, P = 1.06e-07; U87, TMZ vs TMZ + 6-MP, P = 4.33e-11.

k Quantification of TUNEL were shown. (n = 10 for each group). **, $P < 0.001$. MES28, TMZ vs TMZ + 6-MP, P = 2.31e-12; U87, TMZ vs TMZ + 6-MP, P = 1.19e-10.

l-o Represent immunofluorescence images and the quantification of Flag (indicating O6-mG levels) were shown. (n = 10 for each group). Scale bars, 60 μm.

l Validation of immunofluorescent analysis on recognizing O6-MG. Tumor-bearing mice were treated with or without TMZ. The tumor sections were incubated with Flag-MGMT WT or C145S in the presence or absence of O6-BG (MGMT inhibitor). Scale bars, 60 μm. TMZ + MGMT WT vs TMZ + MGMT C145S, P = 2.17e-15; TMZ + MGMT C145S vs TMZ + O6-BG + MGMT C145S, P = 1.49e-15.

m-o Tumor-bearing mice were treated with TMZ together with or without 6-MP. The tumor sections (**m**), tumor adjacent tissues (**n**), and normal brain tissues (**o**) were incubated with Flag-MGMT C145S for immunofluorescent analyses using Flag antibody (indicating O6-mG levels). **m** TMZ vs TMZ + 6-MP, P = 4.1e-13; **n** TMZ vs TMZ + 6-MP, P = 0.25; **n** TMZ vs TMZ + 6-MP, P = 0.16.

Source data are provided as a Source Data file.

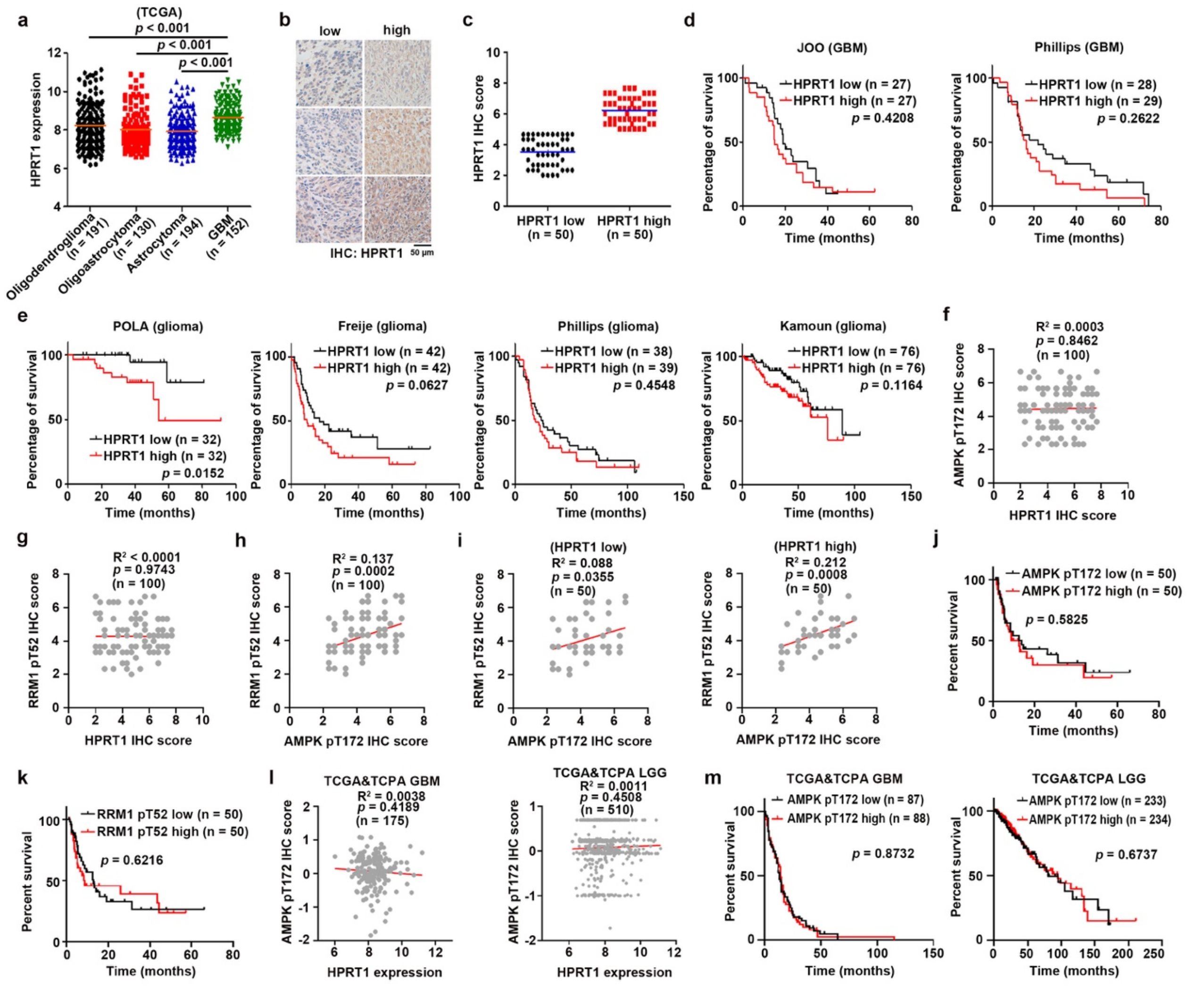

Supplementary Fig. 12 HPRT1 expression predicts poor prognosis of GBM patients

a HPRT1 mRNA expression in different histologic samples was analyzed using the TCGA dataset. Oligodendroglioma vs GBM, P = 4.47e-05; Oligoastrocytoma vs GBM, P = 1.64e-09; Astrocytoma vs GBM, P = 4.93e-16.

b Representative IHC images of HPRT1 staining of clinical GBM sections. (n = 50 for each group).

c Scatterplot of the semiquantitative IHC staining score for HPRT1 in 100 GBM samples.

d Kaplan-Meier survival analysis based on HPRT1 expression from the indicated GBM datasets.

e Kaplan-Meier survival analysis based on HPRT1 expression from the indicated glioma datasets.

f-i Correlation among HPRT1, AMPK pT172, and RRM1 pT52 in primary GBM samples. Note that one dot may represent multiple samples.

j, k Kaplan-Meier survival analysis based on AMPK pT172 and RRM1 pT52 expression from primary GBM samples.

l The Pearson correlation test was used to analyze the correlation between HPRT1 and AMPK pT172 from TCPA and TCGA datasets.

m Kaplan-Meier survival analysis based on AMPK pT172 expression from TCPA and TCGA datasets. Statistics: **a** unpaired Student's t test for two-group comparison. **d-e, j-k, m** Log-rank test for two-group comparison. **f-i** Two-sided Student's t test for two-group comparison. Source data are provided as a Source Data file.

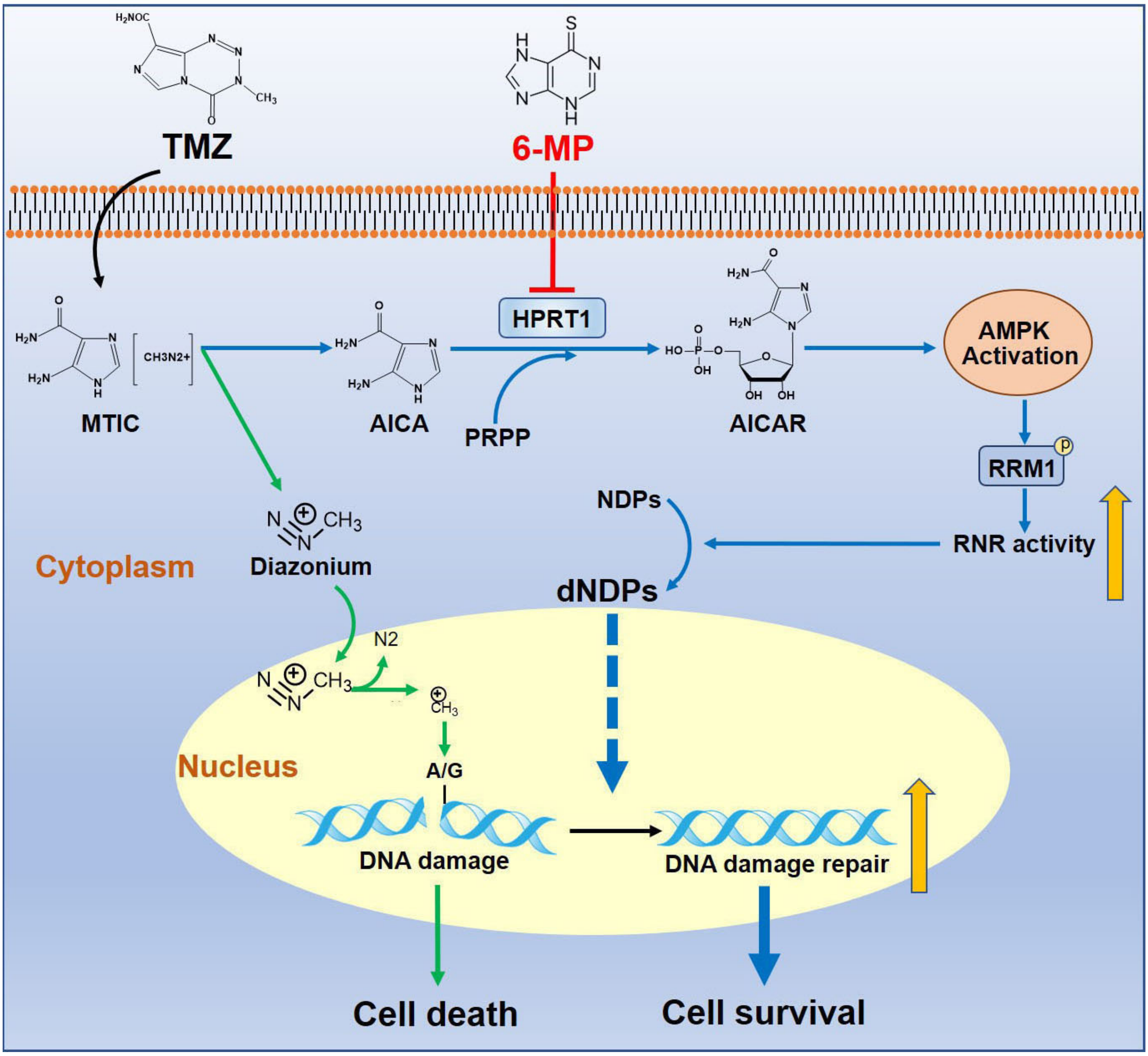

Supplementary Fig. 13 Working model of this study

TMZ is metabolized into methyldiazonium cations and AICA in physiological conditions. TMZ-derived methyldiazonium induces DNA damage and cell death. The other intermetabolite of TMZ, AICA, is converted into AICAR by HPRT1, which results in activation of AMPK and the subsequent AMPK-mediated phosphorylation of RRM1 at T52. This phosphorylation increases RNR activity and promotes the production of dNDPs, which fuels the repair process of DNA damage induced TMZ-derived methyldiazonium. This model illustrates an unknown shield and spear paradox of TMZ which leads to chemoresistance in GBMs.

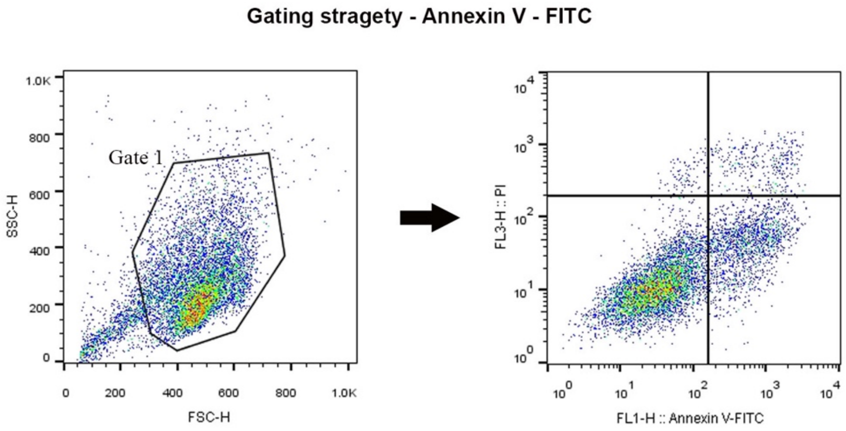

Supplementary Fig. 14 Flow cytometry gating strategy for Annexin V-FITC

Cell-sized particles in Gate 1 were selected for further analysis of populations stained with annexin V-FITC/propidium iodide.

Supplementary Table 1 Identification of T52 phosphorylation of RRM1 by MS

MS/MS（m/z：1214.59）

**Source: RRM1**	**RIR1_ HUMAN**			
**Product ion sequence**	**m/z (cal)**	**m/z (exp)**	**Position**	**b/y ions**
SGV*TTVELDTL	1214.57	1214.62	T52	precursor
GV*TTVELDTL	1127.54	1127.59	T52	y10
SGV*TTVELDT	1083.47	1083.52	T52	b10
V*TTVELDTL	1070.52	1070.57	T52	y9
SGV*TTVELD	982.42	982.46	T52	b9
*TTVELDTL	971.45	971.49	T52	y8
TTVELDTL	891.47	891.51		y_8^0
SGV*TTVEL	867.39	867.43	T52	b8
TVELDTL	790.42	790.45		y_7^0
SGV*TTVE	754.31	754.34	T52	b7
VELDTL	689.37	689.40		y_6^0
SGV*TTV	625.27	625.30	T52	b6
ELDTL	590.31	590.33		y_5^0
SGV*TT	526.20	526.22	T52	b5

MS/MS analysis of precursor ion (m/z 1026.45, phosphorylated RRM1 peptide 49-59) was shown, indicating that T52 was phosphorylated (right). “*”, phosphorylation.
