# Supplementary material for: Hypoxanthine phosphoribosyl transferase 1 metabolizes temozolomide to activate AMPK for driving chemoresistance of glioblastomas
Source: Nat Commun. 2023 Sep 22;14:5913. doi: 10.1038/s41467-023-41663-2 (PMC10516874; doi:10.1038/s41467-023-41663-2)
Supplement: Supplementary file 2 — Reporting Summary [file 41467_2023_41663_MOESM2_ESM.pdf]

nature portfolio

Corresponding author(s): Yongping You, Zhimin Lu, and Xu Qian

Last updated by author(s): NCOMMS-22-44719

Reporting Summary

Nature Portfolio wishes to improve the reproducibility of the work that we publish. This form provides structure for consistency and transparency in reporting. For further information on Nature Portfolio policies, see our Editorial Policies and the Editorial Policy Checklist.

Statistics

For all statistical analyses, confirm that the following items are present in the figure legend, table legend, main text, or Methods section.

n/a	Confirmed	
[ ]	[x]	The exact sample size (*n*) for each experimental group/condition, given as a discrete number and unit of measurement
[ ]	[x]	A statement on whether measurements were taken from distinct samples or whether the same sample was measured repeatedly
[ ]	[x]	The statistical test(s) used AND whether they are one- or two-sided<br>*Only common tests should be described solely by name; describe more complex techniques in the Methods section.*
[x]	[ ]	A description of all covariates tested
[x]	[ ]	A description of any assumptions or corrections, such as tests of normality and adjustment for multiple comparisons
[ ]	[x]	A full description of the statistical parameters including central tendency (e.g. means) or other basic estimates (e.g. regression coefficient) AND variation (e.g. standard deviation) or associated estimates of uncertainty (e.g. confidence intervals)
[ ]	[x]	For null hypothesis testing, the test statistic (e.g. *F*, *t*, *r*) with confidence intervals, effect sizes, degrees of freedom and *P* value noted<br>*Give P values as exact values whenever suitable.*
[x]	[ ]	For Bayesian analysis, information on the choice of priors and Markov chain Monte Carlo settings
[x]	[ ]	For hierarchical and complex designs, identification of the appropriate level for tests and full reporting of outcomes
[ ]	[x]	Estimates of effect sizes (e.g. Cohen's *d*, Pearson's *r*), indicating how they were calculated

Our web collection on statistics for biologists contains articles on many of the points above.

Software and code

Policy information about availability of computer code

Data collection	BD FACSDiva software, Thermo Scientific Vanquish liquid chromatography (LC) system, Q Exactive Mass Spectrometer, ScanScope XT scanner
Data analysis	XCalibur v3.1.66.10, GraphPad Prism 8, Excel

For manuscripts utilizing custom algorithms or software that are central to the research but not yet described in published literature, software must be made available to editors and reviewers. We strongly encourage code deposition in a community repository (e.g. GitHub). See the Nature Portfolio guidelines for submitting code & software for further information.

Data

Policy information about availability of data

All manuscripts must include a data availability statement. This statement should provide the following information, where applicable:

- Accession codes, unique identifiers, or web links for publicly available datasets
- A description of any restrictions on data availability
- For clinical datasets or third party data, please ensure that the statement adheres to our policy

Provide your data availability statement here.

Research involving human participants, their data, or biological material

Policy information about studies with human participants or human data. See also policy information about sex, gender (identity/presentation), and sexual orientation and race, ethnicity and racism.

Reporting on sex and gender	Information on patients' sex was collected based on self-reporting. Patient group has been designed to be sex-homogeneous and sex-based analyses were not included in this study.
Reporting on race, ethnicity, or other socially relevant groupings	Characteristics of the socially relevant grouping (e.g. race) were not collected in this study
Population characteristics	Characteristics of the patients (e.g. age) were not collected in this study.
Recruitment	Primary and recurrent GBM clinical samples were obtained from the First Affiliated Hospital of Nanjing Medical University. And the signed informed consent were obtained from all patients.
Ethics oversight	The use of clinical specimens was approved by the medical ethics committee of the First Affiliated Hospital of Nanjing Medical University.

Note that full information on the approval of the study protocol must also be provided in the manuscript.

Field-specific reporting

Please select the one below that is the best fit for your research. If you are not sure, read the appropriate sections before making your selection.

[x] Life sciences [ ] Behavioural & social sciences [ ] Ecological, evolutionary & environmental sciences

For a reference copy of the document with all sections, see nature.com/documents/nr-reporting-summary-flat.pdf

Life sciences study design

All studies must disclose on these points even when the disclosure is negative.

Sample size	We estimated the sample size considering no significant variation within each group of data. The principle of using the smallest sample size possible was adopted in planning the animal experiments. We estimated the sample size in order to detect a difference in averages of 2 standard deviations at the 0.05 level of significance with an 80% power.
Data exclusions	No data were excluded from any analysis included in our results.
Replication	The experiments were repeated as described in the figure captions, achieving overlapping results, all reported in the figures of the manuscript.
Randomization	Experimental mice were randomized into the experimental groups following tumor inoculation before TMZ or 6-MP treatment according to bioluminescence tumor value to ensure equal mean tumor burden per condition at the start of treatment. For studies not involving animals, no randomization was required.
Blinding	For in vivo studies, blinding were performed only at the start of the experiments, when mice were randomized into the experimental groups following tumor inoculation before TMZ or 6-MP treatment. Moreover, the biolumiscence data were collected in a blinding manner. For all the other subsequent steps, blinding was not feasible given the study design and the labeling requirements for the operative procedures of the facilities. IHC stainings were performed in blinding manner.

Reporting for specific materials, systems and methods

We require information from authors about some types of materials, experimental systems and methods used in many studies. Here, indicate whether each material, system or method listed is relevant to your study. If you are not sure if a list item applies to your research, read the appropriate section before selecting a response.

Materials & experimental systems

n/a	Involved in the study
☐	☒ Antibodies
☐	☒ Eukaryotic cell lines
☒	☐ Palaeontology and archaeology
☐	☒ Animals and other organisms
☒	☐ Clinical data
☒	☐ Dual use research of concern
☒	☐ Plants

Methods

n/a	Involved in the study
☒	☐ ChIP-seq
☐	☒ Flow cytometry
☒	☐ MRI-based neuroimaging

Antibodies

Antibodies used

WB: ACC1 pS79 (Cell Signaling Technology, 11818, 1:1000),
WB: ACC1 (Cell Signaling Technology, 2676, 1:1000),
WB: AMPKα pT172 (Cell Signaling Technology, 50081, 1:1000),
WB: AMPKα (Cell Signaling Technology, 5831, 1:1000),
WB: AMPKα1 (Cell Signaling Technology, 4148, 1:1000),
IP: AMPKα1 (Cell Signaling Technology, 4148, 1:100),
WB: AMPKβ1 (Cell Signaling Technology, 12063, 1:1000),
WB: γ-H2AX (Cell Signaling Technology, 9718, 1:1000),
WB: α-Tubulin (Cell Signaling Technology, 3873, 1:2000),
WB: Flag (Sigma-Aldrich, F3165, 1:1000),
IP: Flag (Sigma-Aldrich, F3165, 1:100),
WB: MGMT (Abcam, ab108630, 1:1000),
WB: UPRT (Abcam, ab251653, 1:1000),
WB: APRT (Proteintech, 21405-1-AP, 1:1000),
WB: OPRT (Proteintech, 14830-1-AP, 1:1000),
WB: QPRT (Proteintech, 25174-1-AP, 1:1000),
WB: HPRT (Proteintech, 15059-1-AP, 1:1000),
WB: RRM1 (Proteintech, 10526-1-AP, 1:1000),
IP: RRM1 (Proteintech, 10526-1-AP, 1:100).

Validation

Rabbit polyclonal antibodies against RRM1 T52 phosphorylation (RRM1 Pt52) was produced by Affinity Biosciences LTD (Cincinnati, OH, USA). A peptide containing RRM1 T52 phosphorylation was injected into rabbits. The rabbit serum was collected and sequentially purified using an affinity column conjugated with non-succinated and phosphorylated RRM1 T52 peptide, respectively, to exclude the antibodies recognizing non-T52- phosphorylated RRM1, followed by an affinity column conjugated with phosphorylated RRM1 T52 peptide to bind to and purify the RRM1 pT52 antibody.

Eukaryotic cell lines

Policy information about cell lines and Sex and Gender in Research

Cell line source(s)

U87 (HTB-14), T98G (CRL-1690), and LN18 (CRL-2610) cells were obtained from ATCC. U251 (09063001) was purchased from Sigma-Aldrich (Shanghai, China). MES28 and GSC3028 cells were obtained from Professor Xiuxing Wang. U251S, U251T3rd, N3S, N3T3rd cells were constructed by our group as previously described.

Authentication

All cell lines were authenticated by PCR-single-locus-technology (Promega, USA. PowerPlex 21 PCR) analysis in "BMR Genomics s.r.l." (Italy).

Mycoplasma contamination

All cells were confirmed to be negative for mycoplasma by PCR as described in the manuscript after every freeze-thaw cycle and before injection into mice.

Commonly misidentified lines
(See ICLAC register)

In our paper, we have not applied the use of misidentified lines

Animals and other research organisms

Policy information about studies involving animals; ARRIVE guidelines recommended for reporting animal research, and Sex and Gender in Research

Laboratory animals

4-week-old female athymic old nude mice were included in this study.

Wild animals

This study did not involved wild animals.

Reporting on sex

All animals were female

Field-collected samples	This study did not involved field-collected samples.
Ethics oversight	The animal study was approved by Nanjing Medical University Animal Experimental Ethics Committee (IACUC-1908037-1).

Note that full information on the approval of the study protocol must also be provided in the manuscript.

Flow Cytometry

Plots

Confirm that:

- [x] The axis labels state the marker and fluorochrome used (e.g. CD4-FITC).
- [x] The axis scales are clearly visible. Include numbers along axes only for bottom left plot of group (a 'group' is an analysis of identical markers).
- [x] All plots are contour plots with outliers or pseudocolor plots.
- [x] A numerical value for number of cells or percentage (with statistics) is provided.

Methodology

Sample preparation	A total of 1 x 105 U87 and MES28 cells pretreated with or without TMZ were collected and washed twice with ice-cold PBS. Then, 100 µl of 1x binding buffer was used to suspend the cells. Cells were stained with 5 µl of Annexin V-FITC and 5 µl of PI staining solution at room temperature for 10 min.
Instrument	Flow-cytometry analysis was performed using a BD LSRFortessa X-20 cytometer (BD Biosciences, USA) and FACSymphony™ A5(BD Biosciences, USA).
Software	FlowJo_V10
Cell population abundance	For flow cytometry analysis of cell apoptosis, 100000 cells per well or group were digested and performed for the experiments. After digest, staining, washing , and cell flitration, about 10000 cells left at each sample
Gating strategy	live/apoptotic cells were gated by FCC/SCC signal, quadrants to distinguish viable. early and late apoptotic cells were used for Annexin V-FITC/propidium iodide staining. An example of gating strategy is provided in Supplementary information file

- [x] Tick this box to confirm that a figure exemplifying the gating strategy is provided in the Supplementary Information.
