# Supplementary material for: Hypoxanthine phosphoribosyl transferase 1 metabolizes temozolomide to activate AMPK for driving chemoresistance of glioblastomas
Source: Nat Commun. 2023 Sep 22;14:5913. doi: 10.1038/s41467-023-41663-2 (PMC10516874; doi:10.1038/s41467-023-41663-2)
Supplement: Supplementary file 3 — Description of Additional Supplementary Files [file 41467_2023_41663_MOESM3_ESM.pdf]

Title: Supplementary Data 1:

Description: List of AMPKα1-associated proteins induced by TMZ treatment

Title: Supplementary Data 2:

Description: Information on HPRT1, AMPK pT172, and RRM1 pT52 IHC scores vs. primary GBM patient survival

Title: Supplementary Data 3:

Description: Information on HPRT1, AMPK pT172, and RRM1 pT52 IHC scores vs. recurrent GBM patient survival

Title: Supplementary Data 4:

Description: Quantification of AICA and AICAR
